# Social dynamics of core members in mixed-species bird flocks change across a gradient of foraging habitat quality

**DOI:** 10.1371/journal.pone.0262385

**Published:** 2022-02-02

**Authors:** Katherine E. (Gentry) Richardson, Daniel P. Roche, Stephen G. Mugel, Nolan D. Lancaster, Kathryn E. Sieving, Todd M. Freeberg, Jeffrey R. Lucas

**Affiliations:** 1 Department of Biological Sciences, Purdue University, West Lafayette, Indiana, United States of America; 2 Department of Wildlife Ecology & Conservation, University of Florida, Gainesville, Florida, United States of America; 3 Department of Psychology, University of Tennessee, Knoxville, Tennessee, United States of America; 4 Department of Ecology and Evolutionary Biology, University of Tennessee, Knoxville, Tennessee, United States of America; MARE – Marine and Environmental Sciences Centre, PORTUGAL

## Abstract

Social associations within mixed-species bird flocks can promote information flow about food availability and provide predator avoidance benefits. The relationship between flocking propensity, foraging habitat quality, and interspecific competition can be altered by human-induced habitat degradation. Here we take a close look at sociality within two ecologically important flock-leader (core) species, the Carolina chickadee (*Poecile carolinensis*) and tufted titmouse (*Baeolophus bicolor*), to better understand how degradation of foraging habitat quality affects mixed-species flocking dynamics. We compared interactions of free ranging wild birds across a gradient of foraging habitat quality in three managed forest remnants. Specifically, we examined aspects of the social network at each site, including network density, modularity, and species assortativity. Differences in the social networks between each end of our habitat gradient suggest that elevated levels of interspecific association are more valuable in the habitat with low quality foraging conditions. This conclusion is supported by two additional findings: First, foraging height for the subordinate Carolina chickadee relative to the tufted titmouse decreased with an increase in the number of satellite species in the most disturbed site but not in the other two sites. Second, the chickadee gargle call rate, an acoustic signal emitted during agonistic encounters between conspecifics, was relatively higher at the high-quality site. Collectively, these results suggest an increase in heterospecific associations increases the value of cross-species information flow in degraded habitats.

## Introduction

Broad foraging niches and plastic foraging behavior often facilitate the use of adaptive foraging strategies when animals face shifting resource availability and novel environments [1–3]. Foraging niche expansion is also facilitated by an increase in social behavior, for example when flocks of seabirds and schools of predatory fish increase foraging conditions for one another (e.g. [4]) or when species in mixed species bird flocks with different specialized roles influence foraging conditions for all species in the flock [5,6]. This is best exemplified in mixed-species bird flocks: individuals that flock with other species experience foraging benefits, such as when intra- and inter-specific associations promote information flow about novel food sites to the point where some species play a keystone role in flock composition and flock functionality [7]. In addition, mixed-species flocks provide predator avoidance benefits that can further improve foraging efficiency [4,8]. For instance, enhanced information flow regarding predation risk can increase the likelihood of predator detection and reduce detection latency, allowing for decreased vigilance rates and increased foraging time [9,10].

Nonetheless, participation in mixed-species flocks can come at a cost when foraging-niche overlap between species intensifies interspecific competition among flock members [6,11,12]; but see [13–15]. Ultimately, the tradeoff between interspecific competition and flocking benefits depends on the degree of foraging niche segregation within a flock [15,16], as well as the abundance and distribution of food, and predation risk [8,14,17]. For this reason, mixed-species flocking typically occurs when food availability is especially low or predation risk is especially high [8,18]. As such, mixed-species bird flocks in temperate climates tend to form during the winter season when food is scarce [19].

However, human-induced habitat degradation can influence or even disrupt the extent of spatial cohesion and individual membership in mixed-species flocks (“social dynamics”: [20–23]. Previously, we showed that in relatively poor quality foraging habitat: (1) core (or leader) flock species expanded their use of space, and (2) satellite (or follower) flock species continued to flock with core flock species into the breeding season [24]. Increased space use may result from an increased need to expand access to foraging habitat [1,25], which in turn increases the requirement for broad-scale information flow. Information flow will depend critically on the social relationships between flock members, which in some respects depends on the type and strength of associations among core individuals (see [26–28].

Here, we describe levels of sociality between two core species, the Carolina chickadee (Family Paridae; *Poecile carolinensis*) and tufted titmouse (Family Paridae; *Baeolophus bicolor*), across a foraging habitat gradient to better understand how degradation of the quality of foraging habitat affects social network structure between ecologically important flock-leader species. Carolina chickadees and tufted titmice occur within temperate deciduous forests in the eastern United States. As core species, they attract satellite (or follower) species, including white-breasted nuthatches (Family Sittidae; *Sitta carolinensis*) and downy woodpeckers (Family Piccidae; *Dryobates pubescens*). These satellite species benefit from mixed-species flocking by copying the foraging locations of the core species [29]; however, their participation in cooperative predator mobbing events also affords antipredator benefits to the flock [30–32]. Within this flocking system, these two satellite species are bark/trunk feeders and the core species forage closer to the canopy [33–35]. The comparatively greater foraging niche overlap between the core species suggests interspecific competition is greater between heterospecific core members than between core and satellite members [15]. Indeed, a heightened competitive climate explains the tendency for chickadees and titmice either to reduce their tendency to flock together unless accompanied by satellite species [24], or to exhibit habitat niche shifting behavior (e.g., Carolina chickadees forage higher in the presence of tufted titmice than in their absence, and the difference in foraging height is positively correlated with the number of titmice present; [31,36].

We hypothesize that one of the adaptive responses to habitat degradation is increased levels of heterospecific association among core members. Our hypothesis (hereafter, the Information and network-cohesion Hypothesis) presupposes that poor-quality foraging habitat would select for reduced aggression and an increase in the number of heterospecific interactions. This hypothesis is based on three assumptions that are valid in the system we studied. One assumption is that heterospecific interactions are favored when intra-specific competition is stronger than inter-specific competition [31] or when subordinate species specifically benefit from the presence of dominant species in disturbed habitats [37]. Moreover, low quality habitats can select for a lack of territorial defense [25,38] which would cause an overall reduction in aggressive interactions. The hypothesis also assumes that key species that facilitate the formation of mixed-species assemblages are not missing due to extreme forest degradation or fragmentation [8,39]. This latter assumption is particularly valid in low-diversity communities such as the system we describe here. In line with this hypothesis, we predict that individuals in relatively poor quality habitat would associate with a wide range of both conspecific and heterospecific individuals, which could maximize information transfer under suboptimal foraging conditions [8,40]. Likewise, we predict a reduced tendency for core species to preferentially associate with conspecifics in order to reduce competition for limited resources [10,41]. Finally, we assume that a decreased distance between flock members improves information flow [42], and thus we predict that the relative height of individuals of the core species within a mixed-species flock will depend on foraging habitat quality. In particular, we predict a reduced foraging height of chickadees relative to titmice in poor quality foraging habitat compared to relative heights observed between individuals found in higher quality habitat, given that individuals in subordinate species of the family Paridae often forage higher than dominant species of the family Paridae [31]. We test our predictions by comparing interactions of free ranging wild birds across a gradient of foraging habitat quality in three managed forest remnants.

## Methods

### Description of study site locations

We conducted our research within a remnant of temperate deciduous forest embedded in an agriculture-dominated landscape in the glaciated region of west-central Indiana. We chose study site locations in three sections of forest managed by Purdue University: Ross Biological Reserve (40.4086°N, 87.0647°W; 37.61 ha), Martell Forest (40.43955°N, 87.03302°W; 38.72 ha), and Stephens Forest (40.6824°N, 86.6276°W; 30.04 ha). Each section contained a unique subpopulation of birds based on the relative distances juvenile parids typically disperse (e.g. [43]. The Ross Biological Reserve and Martell Forest study sites were separated by a distance of 3.53 km. The distance between the Stephens Forest and Ross Biological Reserve study sites was 48.7 km; the distance between the Stephens Forest and Martell Forest study sites was 45.2 km.

We rank the three study sites according to their apparent foraging quality as indexed by levels of energetic deficits (see [24]. The energetic deficits included fat scores, foraging time budgets, home range size, survivorship, and number of offspring. In summary: The fat scores were significantly lower at Stephens Forest but not significantly different between Martell Forest and Ross Biological Reserve. The foraging time budgets were significantly lower at Ross Biological Reserve, intermediate at Martell Forest and highest at Stephens Forest. The home range size at Stephens Forest was significantly larger than those of Ross Biological Reserve. The survivorship was significantly higher in Martell Forest compared to Stephens Forest. Finally, the titmice offspring count was significantly lower at Stephens and Martell Forests compared to Ross Biological Reserve. We therefore qualitatively rank Stephens Forest as the lowest quality site and the Ross Biological Reserve as the highest quality site.

The difference in foraging quality may be related to forestry practices at each site [24]. The Ross Biological Reserve (hereafter ‘high-quality site’) is managed for biological diversity and has been left relatively untouched since its establishment in 1949 [44]. In contrast, Martell Forest (hereafter ‘mid-quality site’) is managed for invasive species control and the following practices are ongoing: cut stump treatments, prescription burns, and application of basal or foliar chemical sprays. The third site, Stephens Forest (hereafter ‘low-quality site’) is a managed timber harvest property that includes three different types of plantation: three walnut stands (*Juglans nigra*; cumulative area of 8.30 ha), one red oak stand (*Quercus rubra*; 0.53 ha), and one yellow poplar stand (*Liriodendron tulipifera*; 1.02 ha). The plantations were created in 1972 and were last thinned in 2004. Forest surrounding the plantation stands is managed (via selective harvest) to promote uneven aged growth (cumulative area of 20.19 ha). See [24] for more details on forest vegetation structure.

### Field observations

We periodically captured and individually marked a total of 200 Carolina chickadees and 199 tufted titmice primarily in August through mid-October in 2015. The number of marked chickadees ranged from 47–78 per site (78, 75, and 47 at the high, mid, and low-quality sites, respectively), while the number of marked titmice ranged from 54–76 per site (76, 69, and 54 at the high, mid, and low-quality sites, respectively). The same banding procedure was followed for our satellite species, the downy woodpecker and white-breasted nuthatch, though they were trapped less frequently (10–12 downy woodpeckers and 37–49 nuthatches banded per site). Birds were drawn into treadle traps locked open on feeding trays filled with black oil sunflower seed four days before traps were set. On the trapping days a combination of treadle traps and mist nets were used to capture the birds. Different configurations of nets were used: 1 net beside the treadle trap, 2 nets set up parallel to the treadle trap, and 3 nets used in triangle formation around the treadle trap. Each captured bird was given a numbered, aluminum U.S. Fish and Wildlife Service band and a unique color combination of plastic leg-bands. We used strips of colored electrical tape placed over the colored band that extended about 10 mm behind the leg to aid in bird identification. No feeder stands were stocked with seeds outside of the trapping periods. All protocols for handling, banding, and observing animals were approved by the Purdue Animal Care and Use Committee (PACUC no. 1306000883).

Flock scan samples of core and satellite associations, individual height, species count, flock size, and color band identity were conducted at all sites during two nonbreeding/winter seasons from September 1 through February 15 in 2015–2017 (see [45] for a description of seasonal patterns of Carolina chickadees in this area). We previously determined that mixed-species flocking at the low-quality site extended into the breeding season [24]. We therefore also conducted scan samples from February 15th through June 1st in 2016 and 2017 at the low-quality site. The surveys were conducted during weekdays between 0800 h and 1700 h on days without precipitation. We visited sites on a rotating basis so that a site was never sampled on consecutive survey days. The surveys were conducted across sub-area plots that were delineated within each site based on topography, private properties lines, and trails. The order in which each plot was surveyed was randomized at the time of arrival to the site each day. One of two trained technicians spent approximately 45 minutes within each plot looking and listening for mixed-species flocks (flocks were defined as the spatial association of at least two birds who were at most 10 m from the next closest bird and traveling in the same direction). The technicians initially sampled in pairs until their inter-observer reliability for flock size and band combinations was >0.90. Technicians were also trained to estimate heights of 5, 10, 20, and 30 meters and had to meet a threshold of 0.85 inter-observer reliability for height estimates before collecting data on height. They then alternated between themselves in their trips to each site. Temperature was recorded using a Kestrel 3000 handheld weather meter once flocks were located.

Scan samples followed a gambit of the group assumption, which implies that each flocking member is associated with other members of the flock [46]. Scan samples occurred every 3–5 min for 60 min or until the flock was no longer in sight. The scan sampling procedure of a located flock was as follows: waiting five minutes after locating a flock, the technician would identify each bird in the flock by species and band color if the bird was banded. In addition, we recorded the number of birds per species and each bird’s height (m) within the flock. Flock numbers collected while observing a single flock were averaged so that a single flock structure and a single average height was used for each flock observed. The average number of scans per flock was 4.8 ± 3.7SD (range 1–20). We used 2016 nonbreeding season data on associations between banded birds based on our definition of flocking for the social network analysis; birds banded after October 1^st^, 2016 were excluded from the analysis so that we did not underestimate the strength of their relationships within the network (these birds could have been unknowingly sighted prior to their banding, but the association data could not be collected without color bands). We also excluded birds encountered fewer than three times to reduce the likelihood of including transients within our analysis (see [13]. We limited our network analysis to core species because the associations during this timeframe and within these conditions included only one banded satellite member, a white-breasted nuthatch.

Note: Flock sizes provide a relevant context for the structure of a social network. Importantly, we previously showed that flock size at the three sites were not significantly different from each other [24]. The average number of core and satellite birds in winter flocks at our sites was as follows (means ± SD): high-quality site, CACH = 2.49±1.19, TUTI = 1.75±1.58, satellites = 1.10±1.22, total flock size = 5.36±2.82; mid-quality site, CACH = 2.74±1.50, TUTI = 1.88±1.35, satellites = 0.87±1.11, total flock size = 5.49±2.30; low-quality site, CACH = 2.66±1.25, TUTI = 1.5±1.34, satellites = 1.32±1.38, total flock size = 5.50±2.60. The respective percent of chickadees and titmice in each flock that were banded was as follows: 86% and 71% at the high-quality site; 85% and 77% at the mid-quality site; and 86% and 81% at the low-quality site.

### Passive acoustic recording

We used chickadee gargle rates as an index of conspecific aggression at each site. The chickadee gargle call is a complex call emitted specifically during agonistic encounters between conspecifics [47–49] and is thus a useful index of aggressiveness in this species. While chickadees and titmice both have a chick-a-dee call system [50], only the chickadee has a gargle call. We obtained gargle rates through passive acoustic recordings using Wildlife Acoustics SM3 song meters (Wildlife Acoustics Inc., Massachusetts, USA). Five SM3 units recorded at each study site in 2016. We had 10 SM3 units that we rotated between the three sites to obtain two continuous weeks of recordings at each site from January through March. The SM3 units were mounted on trees approximately 2 m from the ground and were separated from one another by at least 100 m within each site.

Daily audio files were saved to internal memory as 16 bit, at a sampling rate of 32 kHz at 24.0 gain. The SM3 units were set to record WAV files on stereo channels using foam windscreen-covered SMM-A1 microphones, which are weatherproof, omnidirectional and have a flat (+/-10 dB) frequency response up to 20 kHz and a sensitivity of -11 ± 4 dB (0 dB = 1V/pa at 1 KHz), with a signal-to-noise ratio of > 68 dB. An internal microphone was attached to the SM3 unit on the left channel, while an external microphone was connected to the right channel via an extension cable. The external microphone was hung facing downward from a thin tree sapling (< 10 cm dia), 2 m from the ground, and 40 m away from the SM3 unit. Each of the external microphones were separated from one another by at least 130 m.

Audio files were manually reviewed by J.R.L. using Adobe Audition CS6 software, v 5.0.2. Spectrograms were generated with a 512 spectral resolution, Blackman-Harris windowing function, and a 90 dB decibel range. Sound files were saved as 32 bit with a sampling rate of 44100. We sampled gargle rates mid-morning (0900 h– 0930 h) and mid-day (1200–1230 h) to diversify the conditions under which we sampled gargle calls. Gargle rates were estimated as number of gargles per 30 min. Note that the density of birds at the high quality site (0.56/ha) was about 14% higher than the density at the mid-quality (0.49/ha) and low-quality sites (0.48/ha; see [24].

### Statistical analysis

#### Social network analyses

We constructed group by individual networks of the core species at each site using individuals as nodes and the Simple Ratio association Index (SRI) as edge weights (see S1 Dataset). In association networks, edges are representative of associations between individuals and connect interacting nodes [51]. SRI is identical to the Jaccard index and indicates the proportion of times two individuals were seen together out of the total number of times each was seen under any circumstances. SRI ranged from 0 (never sighted together) to 1 (always sighted together) [52,53]. We used R statistical software to run social network analyses [54]. We used the get_network() function of the asnipe package to convert association matrices into social networks [55], and the igraph package ‘plot’ function with the default Fruchterman Reingold algorithm to depict each network [56]. See S1 Table for specific statistical software package functions used.

We describe network-level measurements, including size, or the number of individuals within a network, and average path length. Network size simply indicates the sample size for associations at each site; in contrast, average path length suggests the efficiency of information within networks, as it refers to the average shortest distance between all individuals in each network (shortest distance being the path that traverses the minimum number of edges between two nodes) [51]. We also report the number of network components and network density. A network component is defined as a portion of the network in which individuals are mutually interconnected and disconnected from the rest of the network [51], whereas network density is the number of realized associations between individuals relative to the possible number of associations [57]. The implication of network components includes interrupted information flow, while dense networks indicate a greater connectedness and a tendency to generate redundant information [57].

We also examined the extent to which each network exhibited community structure; the identification of communities reveals which flocking members belong to tight-knit groups. We used the modularity index (Q) to quantify the degree of community structure [58–60]. This index measures the proportion of edges that occur within communities, relative to the expected proportion if all edges were the result of random associations within the entire network. We detected the presence of communities through a modularity-optimization method, which allowed us to find the partitions in the network and assign nodes into communities such that Q was maximized.

We tested modularity of the empirical network against randomized networks to see if the pattern of network clustering was significantly different than the expected modularity structure under networks with random associations. We constructed the null model using a group membership swap method [61–63], which controls for the observed sizes of flocks and number of times each bird was seen (see [64]. We used the serial method to generate a P-value, for which we specified 1000 swaps from a single initial matrix. The serial test allows us to compare the empirical test statistic against the distribution of the test statistics calculated after each matrix swap.

Although it was not possible to obtain data on all flocks that occurred during the 2016 nonbreeding season, we accounted for sampling error using a bootstrapping scheme to generate a distribution of modularity values associated with the empirical networks. The bootstrapping procedure involved resampling the observed flocks with replacement and recalculating the modularity value 1000 times. A comparison of boxplots shows the modularity values generated from the bootstrapping procedure are greater than those generated from the group membership swap (randomization) procedure at each site (S1 Fig), confirming that community structure in our networks was greater than expected by chance. We also used bootstrapping to measure our confidence in the community assignments and determined whether our community assignments in the empirical networks were generally robust to sampling error [65]. Specifically, we permuted, or resampled, group membership 1000 times. For both the empirical and resampled networks, we measured assortativity, which indicates the degree to which edges occur between nodes of the same community assignment versus between nodes of different community assignments. We used the assortnet package to calculate a p-value of the observed assortativity index as the probability that the observed assortativity value falls within the distribution of assortativity values from the permuted networks. We used the ggplot package in R to graph the empirical assortativity index and 95% confidence intervals for each site [66] (S2 Fig). We also calculated *r*_*community*_, a measurement of robustness of community assignment (see [64] and [67]. For both the randomization and bootstrapping procedures, we used the same edge_betweenness community detection algorithm that we used on the empirical network.

We examined whether networks exhibit assortativity by species to understand whether conspecifics (e.g. chickadee with chickadee or titmouse with titmouse) tend to associate more than heterospecifics (e.g. titmouse with chickadee). We again calculated the assortativity index and followed the same node-label permutation approach as described above to test the null hypothesis that species do not assort more than expected in a random network. In this case, node-label permutations randomized the node species label while maintaining the inherent structural pattern of the network.

#### Relative height between core members in mixed-species flocks

We used a linear mixed model to analyze the response variable, ‘height of each core member’. The fixed effects included ‘site’ (low-quality, mid-quality, high-quality), ‘time-of-day’ (see below), as well as ‘species’ (i.e., focal species), ‘number of satellite members’, ‘number of heterospecific core members’, and ‘number of conspecific core members’ in a flock. We included the ‘number of satellite members’ in the model structure based on previous findings that the number of satellite species moderated aversion between core species [24]. We excluded a quadratic term for number of satellite species after confirming inclusion of this term did not improve model fit (F_1,1714_ = 1.20, P = 0.274). We accounted for diurnal changes in height by including ‘hour’ as a continuous fixed effect (i.e. hour when the scan sample was conducted; this ranged from 0800–1500). Height of the focal bird was log transformed to ensure a normal distribution of residuals (Proc Univariate in SAS 9.4).

The foraging heights of birds in each flock were averaged over multiple scan samples taken of the flock to minimize pseudoreplication. However, flocks observed on multiple days are also a source of psuedoreplication. The problem is that the flocks are fluid enough that we could include ‘flock identity’ in the statistical models. Instead, we used the distribution of locations for each banded bird to identify three sampling units in each site with a minimal interchange of birds across the units (as in [24]. Flocking activity within each sampling unit was treated as repeated measurements. We used a spatial cluster analysis (Proc FASTCLUS in SAS 9.4) of the location of banded birds observed during the flock scan samples to identify the sampling units (miss-classification < 9%), providing N = 3 independent measures of flock-level characteristics per site. As discussed above, each year (2015/16 and 2016/17) was divided into two time intervals (breeding and non-breeding); dimensions of the three site-specific sections were calculated separately for each year and time interval. We did not break each year into more intervals to ensure that we had a sufficient sample size to identify areas with minimal interchange of birds. The spatial dimensions of each cluster were estimated using discriminant function analysis (Proc DISCRIM in SAS 9.4; as in [24]. See S2 Dataset.

#### Gargle rates

We used Poisson mixed models (log link) to test whether the number of gargle calls in 30-min audio files differed among sites (using Proc GLIMMIX in SAS v9.4). In addition to ‘sites’, we also included ‘month’, ‘time-of-day’, and all 2-way and 3-way interactions in the fixed effect structure. Non-significant interaction terms (P>0.05) were sequentially dropped from the model based on the F value. The subject in the model was SM3 unit nested within site and month. See S3 Dataset.

## Results

### Social network analyses

**Fig 1:**
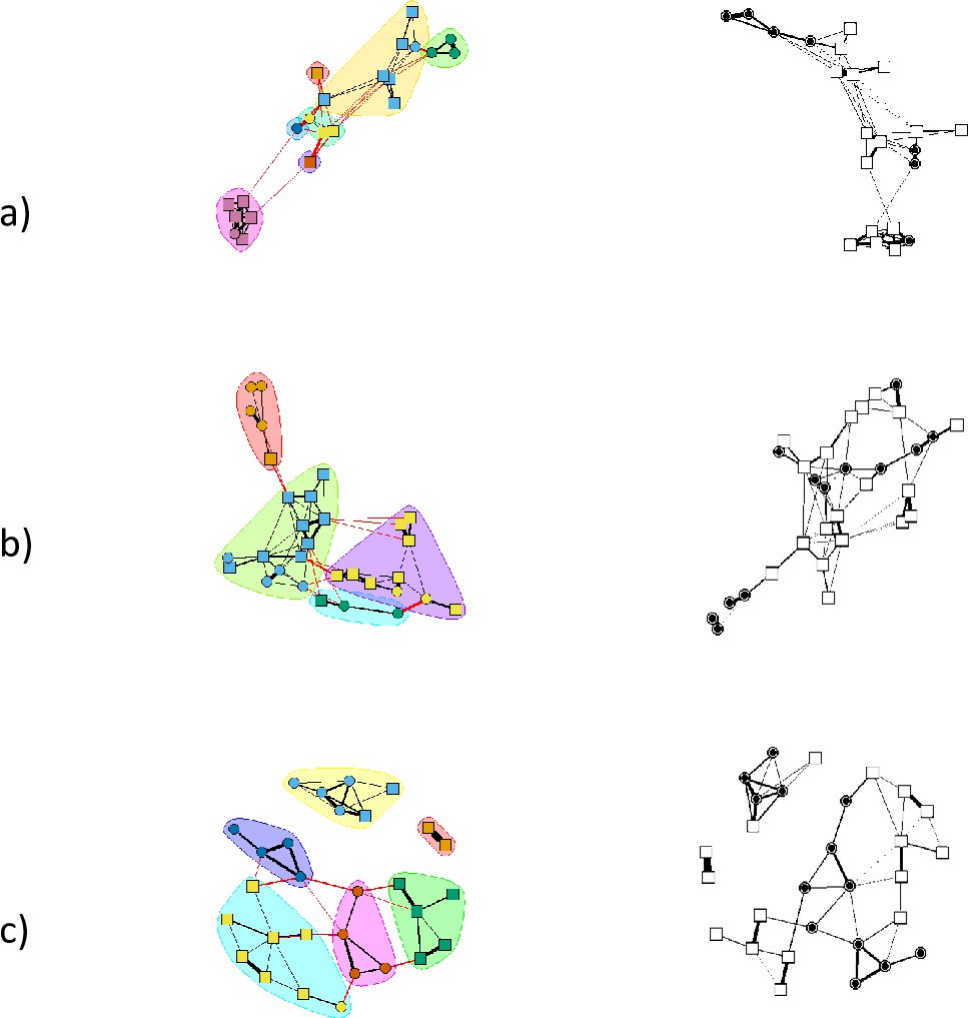
Nonbreeding season social networks of core species (chickadees and titmice) at (a) a low-quality foraging site, (b) a mid-quality foraging site, and (c) a high-quality foraging site. Social community structure (differentiated by bubble color) represents the partitioning of the population into clusters of individuals that flock more often with each other than expected. A non-color coded network is shown to the right of each network for clarity. Square nodes represent chickadees and circular nodes represent titmice.

The network at the mid-quality site was the largest of the three sites’ association sample sizes, with 31 nodes (19 chickadees and 12 titmice) and 65 edges. Network size for the high-quality site was second largest, with 29 nodes (16 chickadees and 13 titmice) and 45 edges. Network size was smallest for the low-quality site, with 22 nodes (15 chickadees and 7 titmice) and 52 edges. Networks generated from the mid-quality and low-quality sites were fully connected as a single component, meaning individuals were mutually interconnected and disconnected from the rest of the network [51]. In comparison, the high-quality network was not fully connected (three unconnected components with sizes of 2, 6, and 21 nodes), which implies interrupted information flow. The average path length (efficiency of information within networks) between two nodes was similar among sites: low-quality site = 2.78; mid-quality site = 2.81; high-quality site = 2.63. However, network density changed inversely with site foraging quality: high-quality site = 0.11, mid-quality size = 0.14, low-quality site = 0.26, meaning the densest network at the low-quality site had a greater connectedness and therefore tendency to generate redundant information [57]. See Fig 1 for depiction of network structure at each site.

Community structure existed across sites, though the number of communities varied as follows: high-quality site: 6; mid-quality site: 4; low-quality site: 7 (Fig 1). The modularity index (Q) was highest at the high-quality site (0.68) but was similar between the mid and low quality sites (0.53 and 0.51, respectively). This result indicates that the tendency for flocking members to form tight-knot groups was highest at the high-quality site. The empirical modularity value was larger than modularity expected in a random network at each of the sites (high-quality: P = 0.002; mid-quality: P = 0.003; low-quality: P = 0.003; see S3 Fig). Each network had large *r*_*community*_ values (Stephens Forest *r*_*community*_ = 0.62; Martell Forest *r*_*community*_ = 0.63; Ross *r*_*community*_ = 0.74), demonstrating our community assignments in the empirical networks were generally robust to sampling error. Significant P-values also confirmed that the empirical assortativity indices fell outside the distribution of assortativity values from the permuted networks (all sites: P < 0.001). Individuals with the same community assignment in the empirical network were usually in the same communities in bootstrap replicate networks and individuals with different community assignments in the empirical network were rarely assigned the same community in resampled networks (S4 Fig), again indicating that our networks are relatively robust.

**Fig 2:**
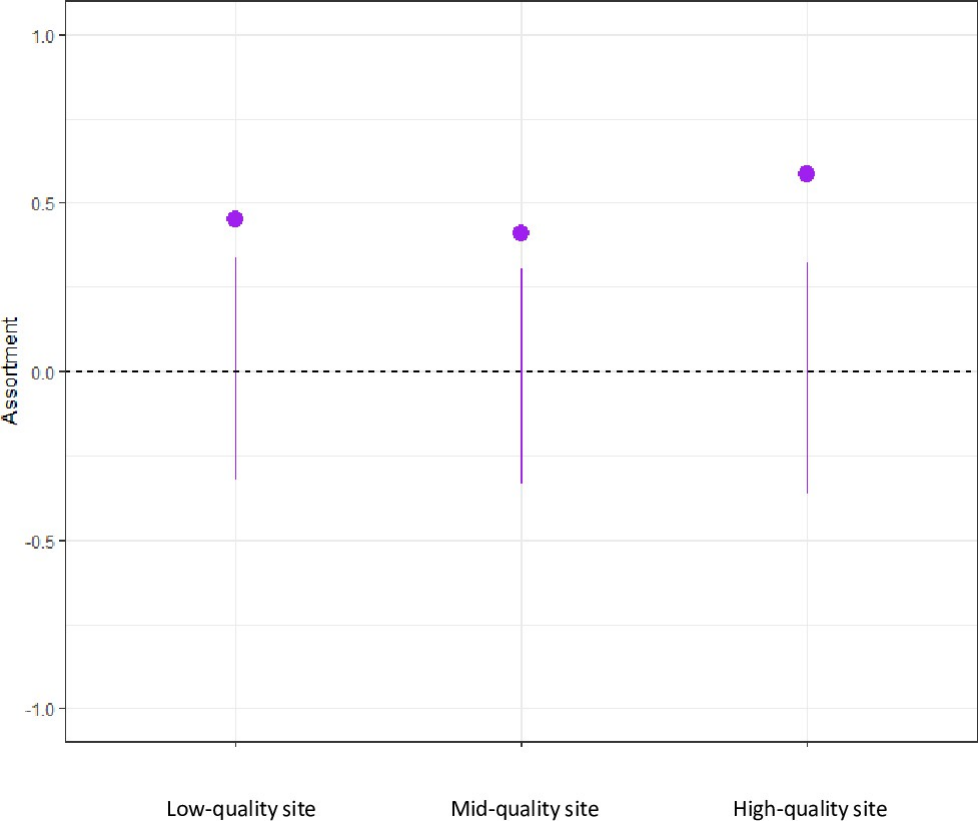
Species assortativity per site (purple dot) and confidence intervals (purple line).

The species assortativity index varied with site quality: high-quality site = 0.56; mid-quality site = 0.41; low-quality site = 0.45. While assortativity was highest at the high-quality site, network permutation confirmed that conspecifics were more likely to associate than heterospecifics in all three networks (Fig 2). Namely, significant P-values confirmed the empirical assortativity indices fell outside the distribution of assortativity values from the permuted networks (high-quality site: P = 0.001; mid-quality site: P = 0.005; low-quality site: P = 0.006; see S5 Fig).

In sum, the network at the low-quality site was more strongly connected, had higher density, and was less modular. In contrast, assortativity of conspecifics was highest at the high-quality site. We now address whether these network properties correlate with the relative height of birds in the flocks and with the use of aggressive calls by chickadees.

### Relative height between core members in the mixed-species flock

**Fig 3:**
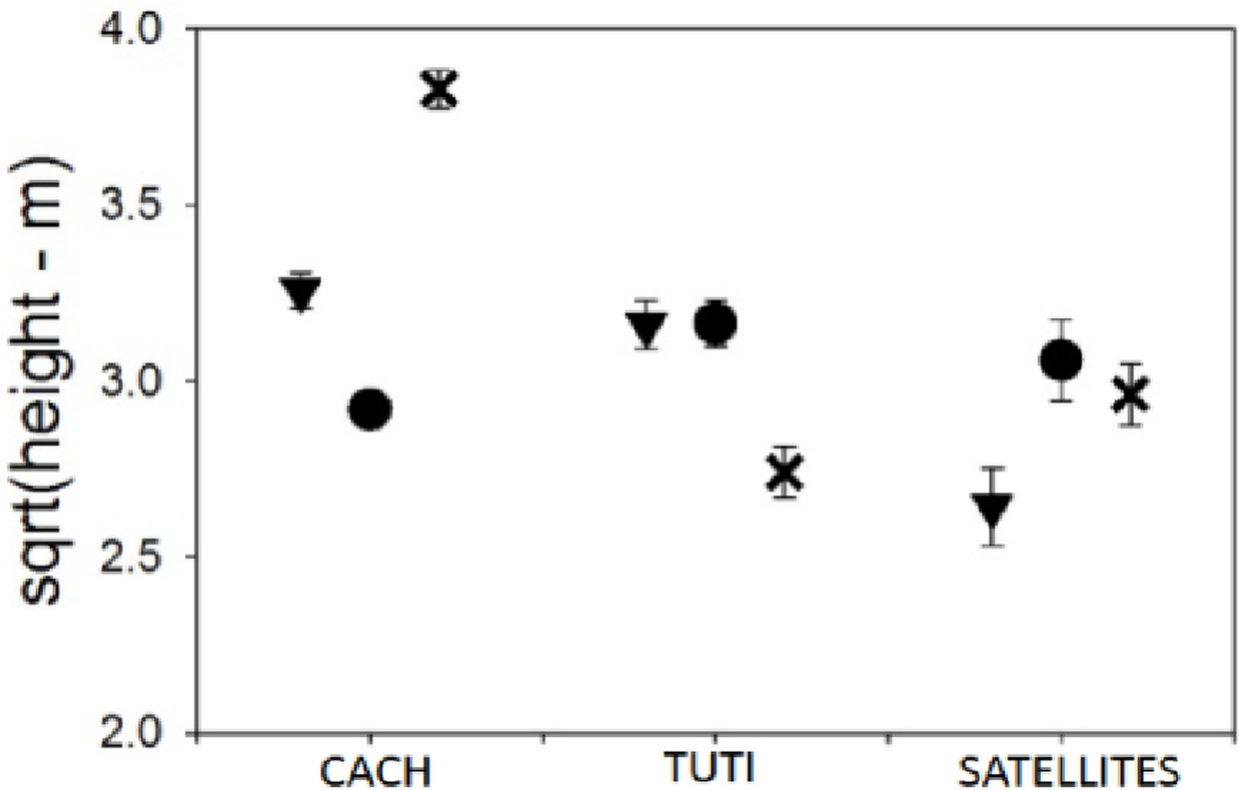
Grand average mean height+/-SE (sqrt transformed) for each group of flock members at each site (error bars are SE). ▼ = least-disturbed site, ● = mid-disturbed site, X = most disturbed site. TUTI: Tufted titmouse. CACH: Carolina chickadee.

**Fig 4:**
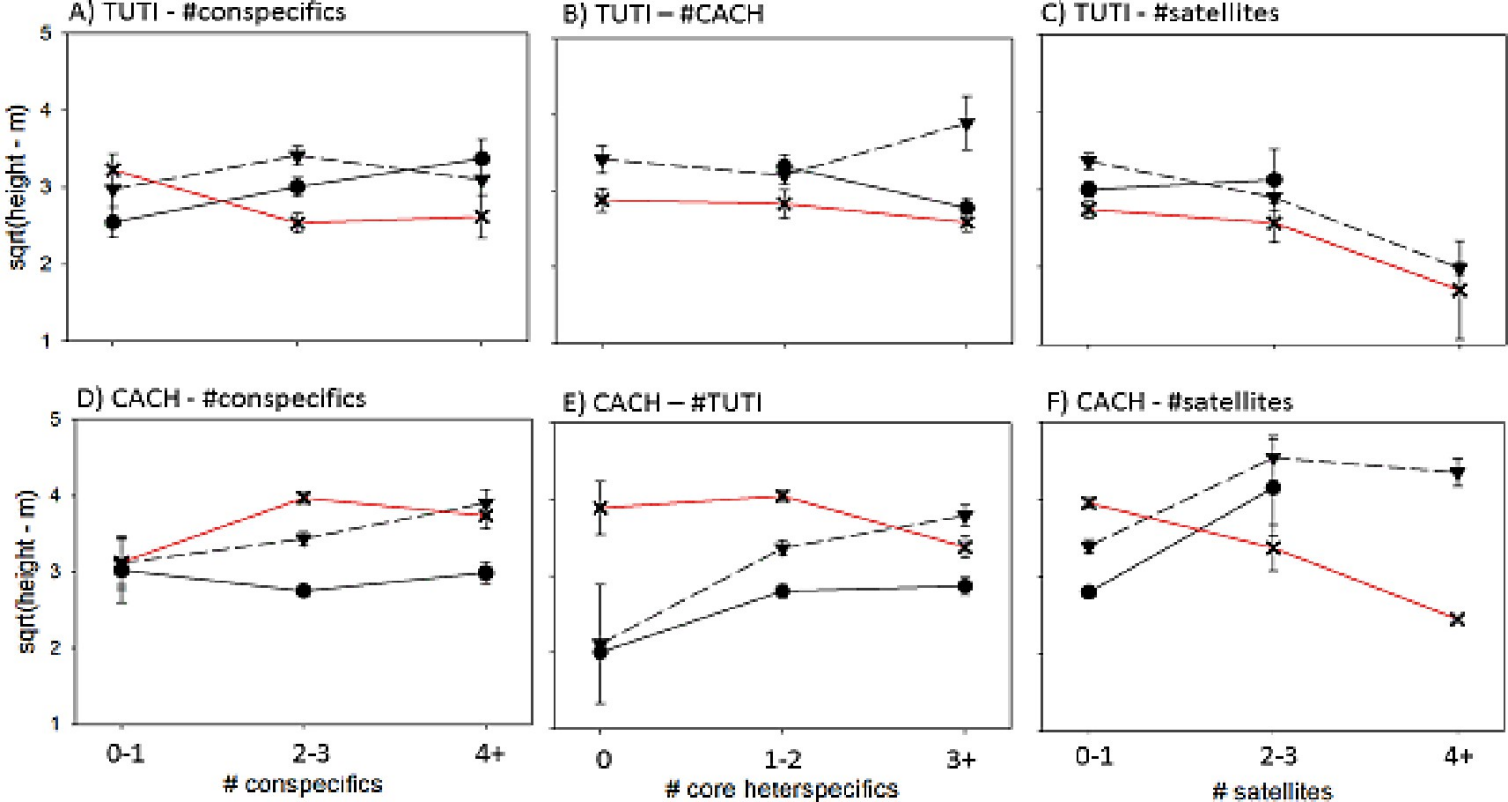
LSMs+/- SE height in flock of tufted titmice and Carolina chickadees in response to the number of conspecifics (A+D), heterospecific core species (B+E) and satellites (C+F) (error bars are SE). ▼ = high-quality site, ● = mid-quality site, X = low-quality site. TUTI: Tufted titmouse. CACH: Carolina chickadee.

Overall, chickadees foraged higher and titmice lower at the most disturbed site compared to the other sites (Fig 3). However, there was a significant four-way interaction between the number of satellite members, site, number of heterospecific members, and core species (Carolina chickadee or tufted titmouse: Table 1). Notably, the height of Carolina chickadees relative to tufted titmice decreased as the number of satellite members increased, but only in the poor foraging-quality site (see Fig 4). We found no similar pattern in the height of titmice (Fig 4). In contrast, the number of conspecifics had no effect on the relative flocking height between core species, regardless of site. We also found a significant time-of-day effect: birds foraged lower in the trees as the day progressed (Tables 1 and S1).

**Table 1:** Type 3 tests of fixed effects for model of relative height between core members in the mixed-species flock.

Effect	Num DF	Den DF	F Value	Pr > F
**Site**	2	15	2.60	0.1073
**Species**	1	15	10.20	0.0060
**# satellites**	1	1692	0.04	0.8451
**# core heterospecifics**	1	1692	20.59	< .0001
**# conspecifics**	1	1692	0.01	0.9351
**Site x Species**	2	15	20.31	< .0001
**# satellites x Site**	2	1692	1.07	0.3437
**# core heterospecifics x Site**	2	1692	1.58	0.2067
**# satellites x Species**	1	1692	6.69	0.0098
**# core heterospecifics x Species**	1	1692	14.56	0.0001
**# satellites x # core heterospecifics**	1	1692	10.05	0.0016
**# satellites x # core heterospecifics x Site**	2	1692	0.21	0.8137
**# satellites x # core heterospecifics x Species**	1	1692	4.18	0.0410
**# core heterospecifics x Site x Species**	2	1692	9.29	< .0001
**# satellites x Site x Species**	2	1692	6.01	0.0025
**# satellites x # core heterospecifics x Site x Species**	2	1692	3.64	0.0266
**Hour**	1	1692	7.26	0.0071

### Gargle rates

**Fig 5:**
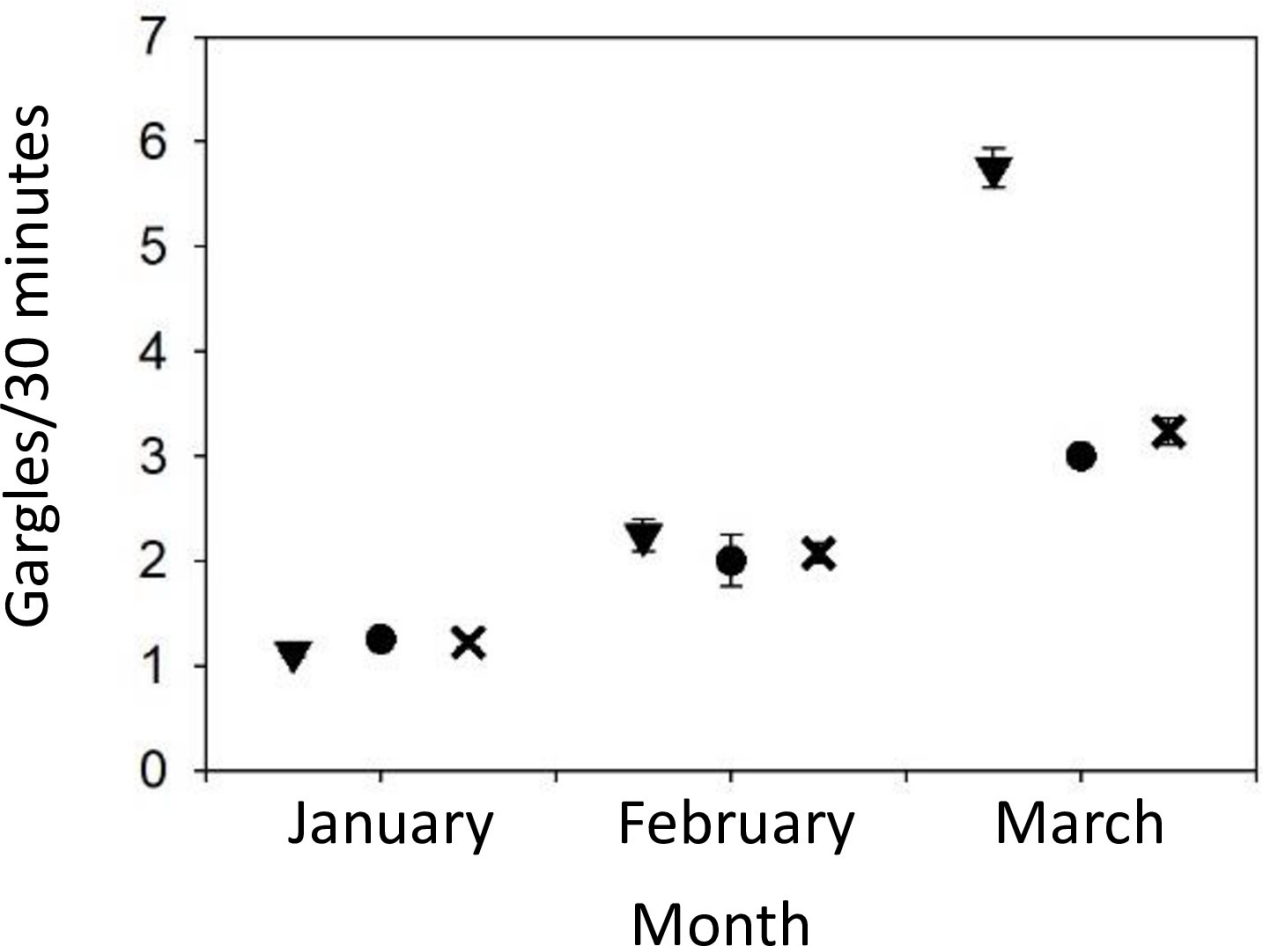
Back transformed least squares means of gargle rates (± standard error bars) across sites and months. ▼ = high-quality site, ● = mid-quality site, X = low quality site.

There was a significant main effect of both site (F_2,19_ = 9, P = 0.002) and month (F_2,19_ = 517.32, P < 0.001) on gargle rates, as well as a significant interaction effect between site and time-of-day (F_2,16_ = 5.62, P = 0.014). At the mid-quality and high-quality sites, least square means (± SE) for gargles rates during the 9–9:30 AM sample (2.02±0.10 and 2.74±0.21, respectively) were relatively higher than during the 12–12:30 PM sample (1.89±0.10 and 2.17±0.08, respectively). In contrast, least square means (± SE) for gargles rates during the 9–9:30 AM sample (1.94±0.07) were relatively lower during the 12–12:30 PM sample (2.10±0.07) at the low-quality site. In all cases gargle rate increased from January through March as expected with the onset of breeding (see Fig 5). However, the site effect results also form a significant interaction effect between location and month (F_4,19_ = 20.22, P < 0.0001). Specifically, gargle rates substantially increased in March at the high-quality site compared to the other two sites (see Fig 5), implying an increased level of aggressiveness in the breeding season at the high-quality site. In short, chickadee aggressive call rates increased significantly more at the high-quality site compared to the other sites.

## Discussion

Our results provide support for the Information and network-cohesion Hypothesis. We observed differences in aggression and network structure characteristics that suggest information transfer facilitated by mixed-species flocking behavior is particularly important in the suboptimal foraging conditions within our habitat gradient. This, along with our previous finding that satellite species continued to flock with core species into the breeding season in a relatively poor-quality foraging habitat [24], suggests that habitat quality degradation increases the value of interspecific associations. Moreover, a decrease in flocking height of Carolina chickadees with increasing numbers of satellites in the low-quality site indicates that interspecific associations may also offer additional benefits in poor foraging conditions. We conclude that heterospecific associations can increase adaptive responses to degradation, such that the benefits of mixed-species flocking outweigh the potential costs of interspecific competition.

### Notable differences in social dynamics across the foraging habitat gradient

While community structure existed at each site, the tendency to form tight-knit groups (network modularity) was highest and network connectedness was lowest at the high-quality site relative to other sites. Preferential association of core species with conspecifics was also greatest at the high-quality site. Collectively, these results suggest that the value of interspecific associations increases in low-quality habitat and aligns with our prediction that core species are more likely to associate preferentially with conspecifics in high quality foraging conditions. In our low-quality habitat, it is likely that weak network modularity and stronger network connectedness aids in broad-scale information flow that is necessary given the expanded home range size of birds in this habitat [24]. Indeed, information about the location of food sources can help reduce foraging-related costs such as locomotory costs, and costs associated with assessing, learning and relocating food supplies [68].

Our social network results align with those reported in other studies conducted in degraded habitat and provide further support for the conclusion that habitat degradation influences mixed-species flocking dynamics [69]. For instance [70], found that network density and social stability increased in fragmented habitats in Sleepy lizards (*Tiliqua rugosa*) [22]. likewise observed stronger ties among red-backed fairy wrens (*Malurus melanocephalus*) where recent burns restricted habitat use. In each case, habitat degradation led to an increase in the overlap of space use among individuals, similar to our finding; however, the mechanism behind the increased overlap differed. In both of these earlier studies, habitat conditions effectively restricted movement. In our case, all sites occurred within forest remnants, but home range size (i.e. space use) varied according to the quality of foraging habitat at each site. Together, these findings suggest that similar changes in social network structure can occur when there is greater overlap in space use in degraded habitat, regardless of whether the overlap is attributable to expanded space use or to restricted movement.

Flock size represents an important additional component to social network structure across our three sites. As we showed previously [24], flock sizes were not significantly different between our sites (see Methods) even though network connectedness was higher at the most disturbed site. This combination of traits suggests that flock dynamics at the most disturbed site follow a fission-fusion flocking pattern (see [71]. The implication is that a reduction in territoriality in the low-quality site resulted in a fission-fusion flocking system where individuals search for food over broader areas, but in so doing interact with a more diverse set of individuals. Alternatively, greater levels of territoriality in our high-quality site generated fairly viscous, tight-knit social networks. These patterns support [71] who showed that spatial environmental variability should enhance fission-fusion dynamics in birds.

Birds in our most disturbed site also spent a greater percent of time foraging compared to birds at the other two sites, and birds at this disturbed site had reduced fat loads in winter [24]. These birds clearly showed a high degree of energetic stress, which likely increased levels of competition. This increased competition could in turn alter an information quality-competition tradeoff, selecting for enhanced sharing of social information among heterospecifics [10]. Thus, the results shown here fit the pattern described by [10], but they also illustrate a potential resolution of a tradeoff between flock size and the level of network connectivity. Low quality habitat appears to constrain flock size but at the same time selects for increased information exchange between individuals. A fission-fusion flock system addresses both of these requirements by allowing the maintenance of limited flock size while facilitating information exchange across a relatively large number of individuals in the social network.

Our finding that the chickadee gargle call rate, an acoustic signal emitted during agonistic encounters between conspecifics [47–49], was significantly higher at the high-quality site relative to the other sites suggests that poor quality foraging habitat also selects for reduced aggression among conspecifics. However, part of this increase could result from a 14% increase in the density of birds at the high-quality site compared to the other two sites. Nonetheless, our data suggest that aggression rates were higher in the spring at the high quality site compared to the other sites based on two facts: (1) gargle rates were identical across all three sites in January and February, and (2) gargle rates increased at the high-quality sites by 154% compared to increases of 50% and 56% at the other two sites, increases substantially greater than the difference in relative bird densities.

It is possible that the differential gargle rates can be explained by the fitness costs associated with conspecific aggression in low quality habitat, resulting in a breakdown in territoriality when within-territory resource levels are insufficient [25]. Indeed, an increase in space use and home range overlap in the poor-quality foraging habitat suggests reduced territoriality among conspecifics [24]. However, the interpretation of this result is further complicated by the fact that we did not evaluate gargle rates beyond March, meaning we cannot rule out the possibility that gargle rates were significantly higher in March because of an earlier onset of the breeding season at the high quality site relative to the lower quality foraging habitats (see [72] for a counter example where habitat quality had no significant effect on timing of breeding). Nevertheless, this finding supports our prediction that foraging habitat influences the social dynamic among flock members.

### Microhabitat foraging niche shift (relative height) and relevance of flock composition

Relative flocking height of chickadees was highest at the most disturbed site both in absolute terms and relative to the other flock mates. However, flocking height decreased at this site with an increase in the number of satellites suggesting that flock composition influences the tradeoff between information value and distance [42]. We hypothesize that the decrease in relative height between core species is a consequence of Carolina chickadees reducing their distance from satellites within the flock. By coming down to flock nearer to satellites, the chickadees would flock closer to tufted titmice, which tend to flock at heights similar to the satellite species. This conditional shift in the core species foraging niche is explained in part by the fact that satellite members of these north-temperate flocks act as low cost, information-rich informers. We assume that: (1) there is minimal overlap in foraging niche, and thus minimal interspecific competition, between these satellite and core species (see [33–35,73] for core species), and (2) the white-breasted nuthatches in particular contribute to information flow while participating in predator mobbing events with the core species [74]. Although valuable information is also gained from flocking closer to ecologically similar heterospecific core members (i.e., information that improves flock coordination, and/or leads to local enhancement or social learning of food type/location and foraging technique), the benefit of this information is countered by costs related to interspecific competition and social dominance ranking within a flocking hierarchy [14]. Indeed, our social network analysis supports the conclusion that core/satellite information flow is especially important in the low-quality habitat. Further research is needed to elucidate whether a greater number of satellite members in mixed-species flocks actually mitigates the competitive costs associated with flocking nearer to heterospecific core members in low quality foraging habitat. Such knowledge would provide key insight into whether satellite members can optimize the value of information obtained through mixed-species flocking.

## Conclusion

This current work, along with [24], offers a case study that provides evidence of how degradation of foraging habitat quality can affect mixed-species flocking dynamics. Our observations of conspecific aggression and network structure of heterospecific core members along a gradient of habitat foraging quality support the Information and network-cohesion Hypothesis, which predicts that heterospecific associations will increase as adaptive responses to habitat degradation. Based on the assumption that information flow decreases with the distance between flock members, the relationship between the number of satellite members and relative flocking height of core species underscores the potential importance of satellite members and their facilitation of information flow between core species. These results suggest that the benefits associated with the transfer of information through mixed-species flocks outweigh the cost of interspecific competition with ecologically similar heterospecific core members. Our findings also suggest that the foraging benefits attributed to mixed-species flocking are oversimplified: satellites may copy the foraging locations of core species, but information flow between core species could be improved by the presence of satellites. From a conservation perspective, this complex flocking dynamic highlights the importance of maintaining flock integrity in terms of core and satellite species composition (see also [75].

It is important to keep in mind that the results from this study and [24] are based on the four primary species found in mixed-species flocks in north temperate forests (see [9,31,76]. The behavioral responses to habitat degradation observed in these flocks may differ from that found in tropical forests (e.g., [77], where seasonal variation in food availability and energetic stress is typically less than in temperate climates [78]. Moreover, the patterns we describe here may result from an increased importance of species-specific associations where species diversity is quite low [23]. We therefore recommend conducting similar studies in the tropics to test the applicability of the Information and network Cohesion hypothesis across climatic zones.

Finally, we were able to describe relevant differences in social dynamics across a disturbance gradient in part because our social networks were constructed based on free-ranging interactions between individuals (also see [79–81]. In contrast, a number of studies of avian networks are based on associations at points of space such as nest sites [82] or co-localization in capture nets [83]. These studies include co-localization on a grid of feeders [41,84–87]. These latter studies in particular have provided a critical understanding of information flow, especially as it relates to novel conditions. However, foraging/predation-risk tradeoffs, and their role in the flow of social information related to that tradeoff, are inevitably lost when the networks are derived from interactions at feeders. Of course, networks derived from free-ranging birds will be less data-rich than those derived from point-specific interactions, but these studies are valuable given the additional understanding of the effect of stress on the structure of social networks.

## Supporting information

S1 Fig Modularity values. Boxplots showing the modularity values generated from the bootstrapping procedure are greater than those generated from the group membership swap (randomization) procedure at each site.
(PDF)

S2 Fig Empirical assortativity indices. Empirical assortativity index and 95% confidence intervals for each site.
(PDF)

S3 Fig Empirical modularity values. Empirical modularity value was larger than modularity expected in a random network.
(PDF)

S4 Fig Bootstrap replicate networks. Consistency among community assignment in the empirical and bootstrap replicate networks indicates social networks are relatively robust.
(PDF)

S5 Fig Empirical assortativity indices compared to assortativity values of permuted networks. P-values confirmed the empirical assortativity indices fell outside the distribution of assortativity values from the permuted networks.
(PDF)

S1 Table Network analysis software functions. Statistical software package functions used in network analyses.
(PDF)

S1 Dataset Core species networks. Association network datasets for the low-quality, mid-quality, and high-quality sites.
(CSV)

S2 Dataset Height. Dataset for relative height between core members in mixed-species flocks analysis.
(CSV)

S3 Dataset Gargle rates. Dataset for garage rate analysis.
(CSV)
