# Supplementary material for: Social dynamics of core members in mixed-species bird flocks change across a gradient of foraging habitat quality
Source: PLoS One. 2022 Feb 2;17(2):e0262385. doi: 10.1371/journal.pone.0262385 (PMC8809581; doi:10.1371/journal.pone.0262385)

S1 Figure. Modularity values generated from the bootstrapping procedure are greater than those generated from the group membership swap (randomization) procedure at each site.

a) High-quality

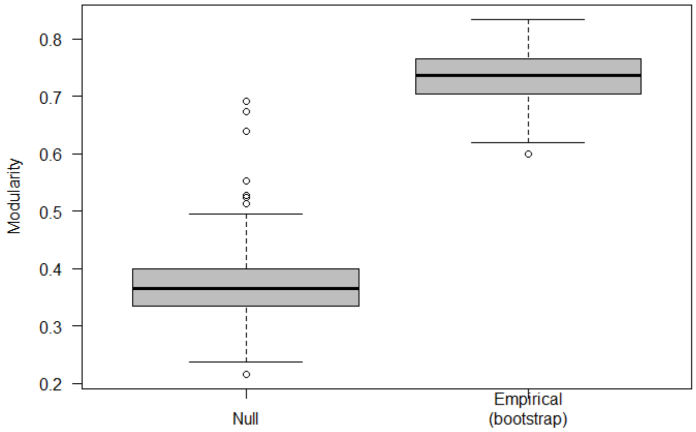

a) Mid-quality

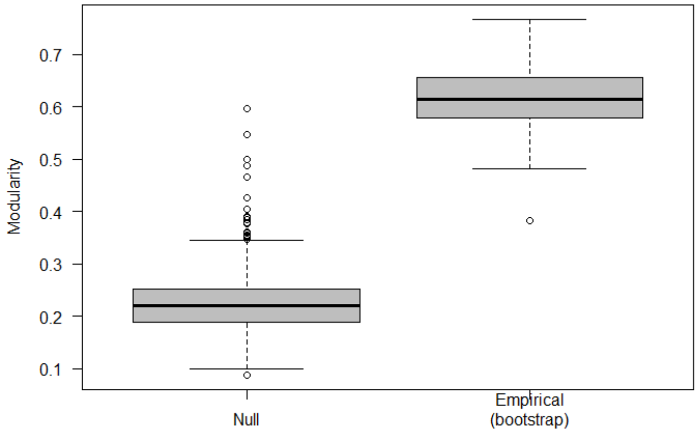

3) Low-quality

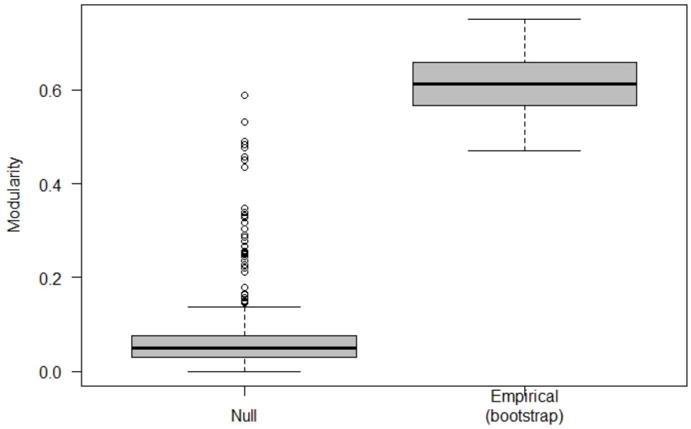

Supplement: S1 Fig — Boxplots showing the modularity values generated from the bootstrapping procedure are greater than those generated from the group membership swap (randomization) procedure at each site. (PDF) [file pone.0262385.s001.pdf]
