# Supplementary material for: Social dynamics of core members in mixed-species bird flocks change across a gradient of foraging habitat quality
Source: PLoS One. 2022 Feb 2;17(2):e0262385. doi: 10.1371/journal.pone.0262385 (PMC8809581; doi:10.1371/journal.pone.0262385)

S2 Figure. Assortativity coefficient for social communities at each site (purple dot) and confidence intervals (purple line).

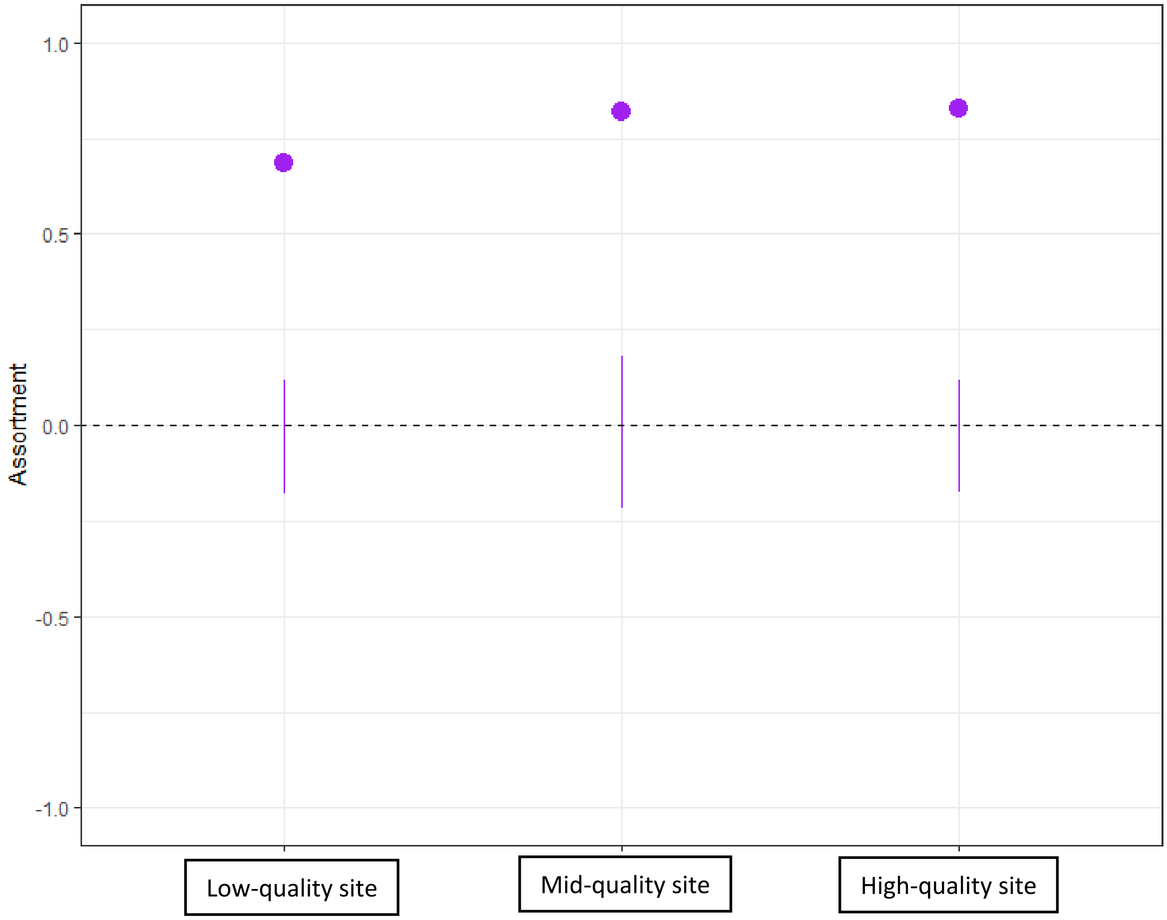

Supplement: S2 Fig — Empirical assortativity index and 95% confidence intervals for each site. (PDF) [file pone.0262385.s002.pdf]
