# Supplementary material for: Social dynamics of core members in mixed-species bird flocks change across a gradient of foraging habitat quality
Source: PLoS One. 2022 Feb 2;17(2):e0262385. doi: 10.1371/journal.pone.0262385 (PMC8809581; doi:10.1371/journal.pone.0262385)

S3 Figure. The observed modularity value (red asterisks) significantly exceeds the expected value for social communities at each site.

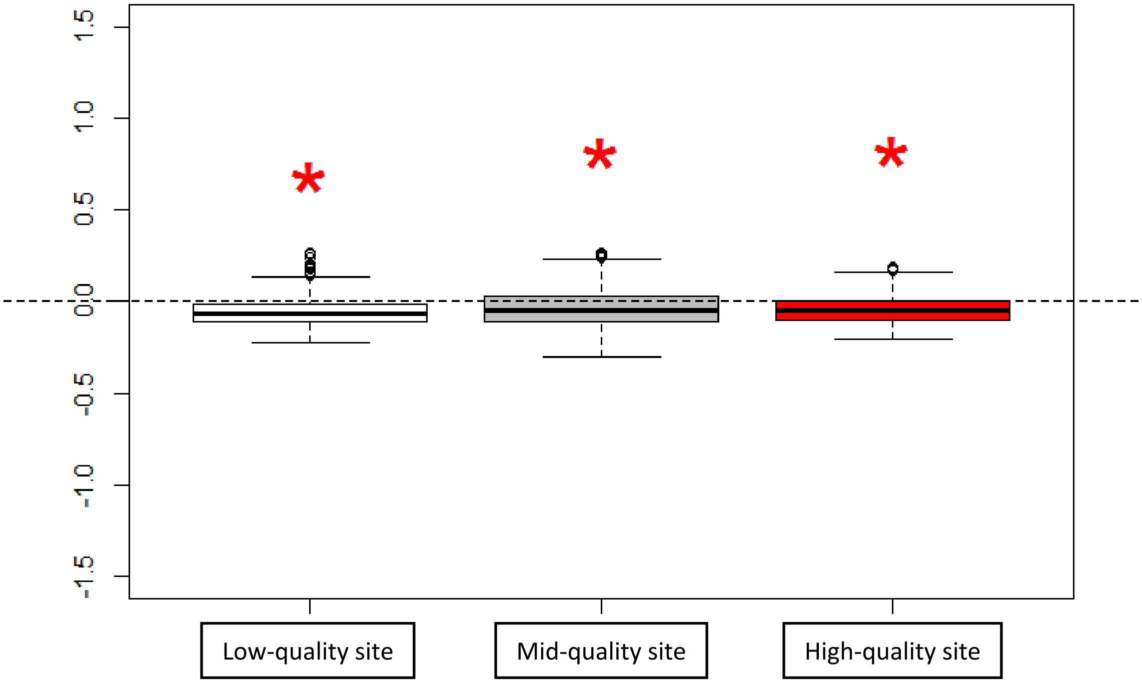

Supplement: S3 Fig — Empirical modularity value was larger than modularity expected in a random network. (PDF) [file pone.0262385.s003.pdf]
