# Supplementary material for: Social dynamics of core members in mixed-species bird flocks change across a gradient of foraging habitat quality
Source: PLoS One. 2022 Feb 2;17(2):e0262385. doi: 10.1371/journal.pone.0262385 (PMC8809581; doi:10.1371/journal.pone.0262385)

S4 Figure. Pairs of individuals in the same community in the empirical network (networks above black line) were usually in the same communities in bootstrap replicate networks (networks below black line).

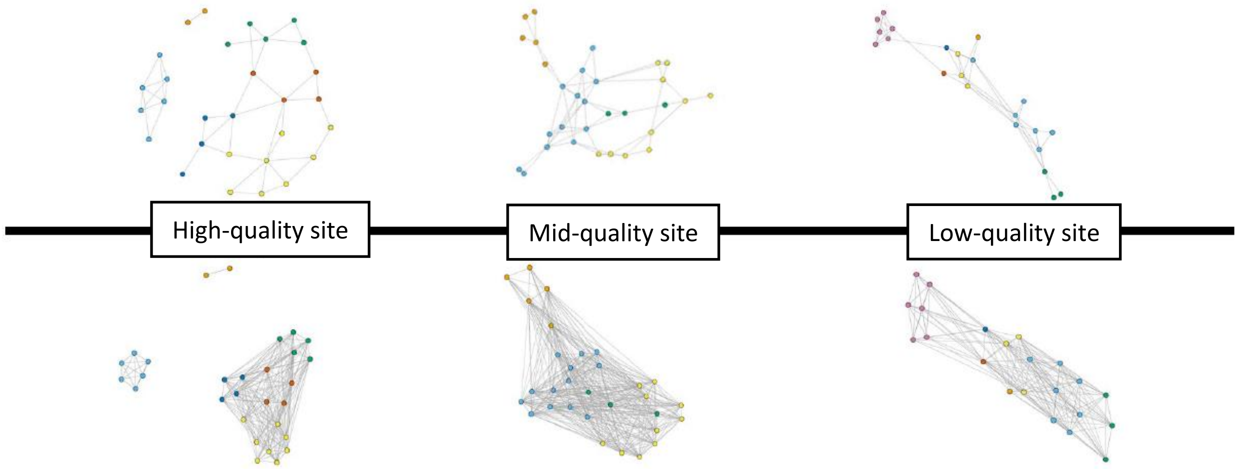

Supplement: S4 Fig — Consistency among community assignment in the empirical and bootstrap replicate networks indicates social networks are relatively robust. (PDF) [file pone.0262385.s004.pdf]
