# Supplementary material for: Social dynamics of core members in mixed-species bird flocks change across a gradient of foraging habitat quality
Source: PLoS One. 2022 Feb 2;17(2):e0262385. doi: 10.1371/journal.pone.0262385 (PMC8809581; doi:10.1371/journal.pone.0262385)

S5 Figure. The observed value (red asterisks) significantly exceeds the expected value of the assortativity index for species at each site.

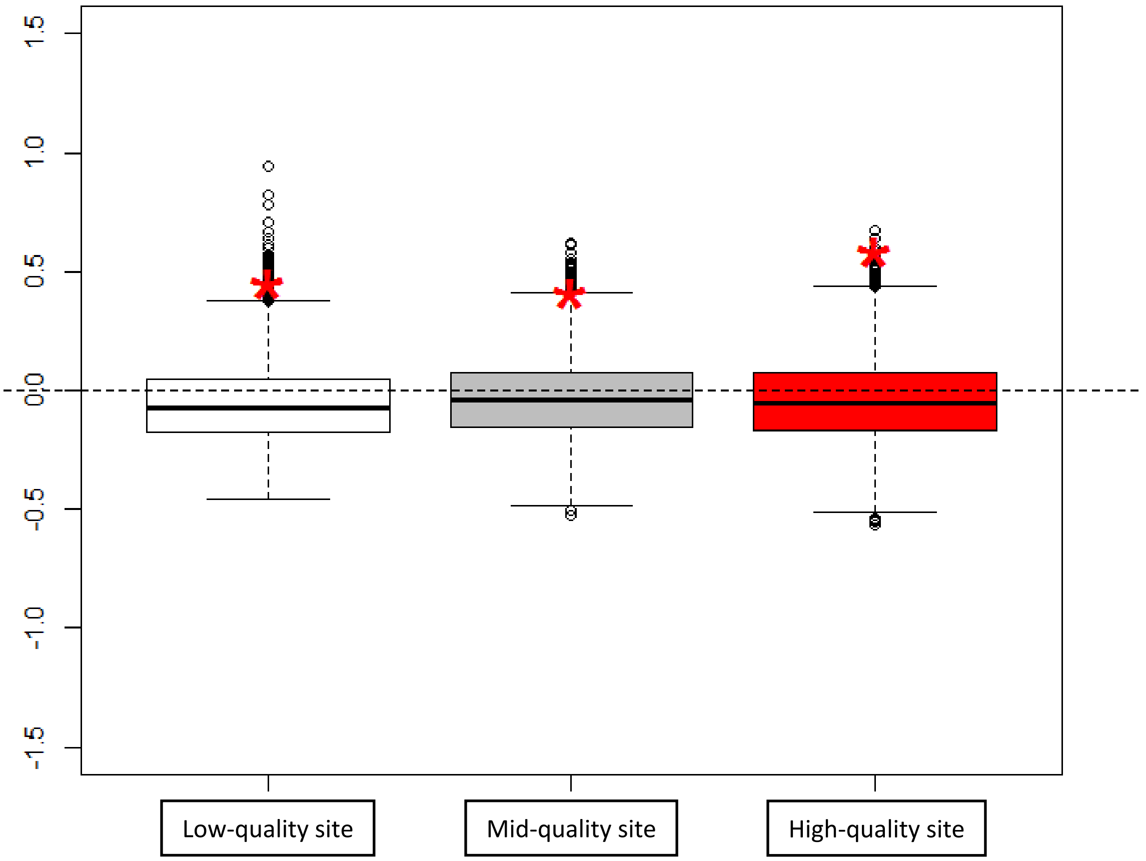

Supplement: S5 Fig — P-values confirmed the empirical assortativity indices fell outside the distribution of assortativity values from the permuted networks. (PDF) [file pone.0262385.s005.pdf]
