# Supplementary material for: Social dynamics of core members in mixed-species bird flocks change across a gradient of foraging habitat quality
Source: PLoS One. 2022 Feb 2;17(2):e0262385. doi: 10.1371/journal.pone.0262385 (PMC8809581; doi:10.1371/journal.pone.0262385)
Supplement: S1 Table — Statistical software package functions used in network analyses. (PDF) [file pone.0262385.s006.pdf]

S1 Table. Description of functions of R statistical software packages used in social network analyses.

Function	Description	Package	Reference
Bipartite_projection	Projection of bipartite graph into two one-mode networks. The resultant weighted adjacency matrix of the bipartite projection is the matrix product of the interaction matrix and its transpose.	igraph	Csardi and Nepusz 2006
Assortment.discrete	Calculates assortativity coefficient, which indicates degree to which edges occur between nodes of the same community assignment versus between nodes of different community assignments.	Assortnet	Farine 2014
Sample()	Random reassignment of each node while otherwise maintaining network structure.	Assortnet	Farine 2014
Edge.betweenness.community	Community detection algorithm that involves a hierarchial decomposition process. Initially, edges are assigned edge-betweeneess scores; lowest scored edges are then removed.	igraph	Csardi and Nepusz 2006; Neman & Girvan 2004; Nerurkar et al. 2019
Mean_distance	Calculation of average path length between nodes, ignoring pairs of nodes in different components.	igraph	Csardi and Nepusz 2006
Edge_density	Calculation of network density.	igraph	Csardi and Nepusz 2006
Transitivity	Calculation of the global clustering coefficient and average local clustering coefficient.	igraph	Csardi and Nepusz 2006
Network_permutation	Group membership swapping algorithm that compares the single empirical value of modularity against a distribution of modularity values generated from randomizing the network.	asnipe	Farine 2013
